# Development and psychometric testing of a caregiver self-efficacy scale for caregivers of individuals with serious mental illnesses

**DOI:** 10.1590/1806-9282.20241551

**Published:** 2025-06-02

**Authors:** Cansu Guler, Nihan Durgu

**Affiliations:** 1 Mugla Sıtkı Koçman University, Faculty of Health Sciences, Department of Psychiatric Nursing, Muğla, Turkey.; 2 Manisa Celal Bayar University, Faculty of Health Sciences, Department of Psychiatric Nursing, Manisa, Turkey.

**Keywords:** Mental disorders, Caregiver, Efficacy, Self, Questionnaire, Validity, Reliability

## Abstract

**OBJECTIVE:**

The aim of this study was to develop a scale to assess the self-efficacy of caregivers of individuals with serious mental illnesses and to determine its psychometric properties.

**METHODS:**

This study used a methodologic, cross-sectional, and descriptive design. A draft of a 26-item scale was developed based on interviews with caregivers and a literature review. The construct validity of the scale was tested by exploratory factor analysis. The reliability of the scale was tested. Caregivers of 348 individuals with serious mental illnesses participated in the study.

**RESULTS:**

The exploratory factor analysis produced a 20-item scale defined by three factors. Reliability coefficient values were adequate.

**CONCLUSION:**

The caregiver self-efficacy scale for caregivers of individuals with serious mental illnesses developed in this study is a valid and reliable instrument that can be used for measuring the self-efficacy of caregivers of individuals with serious mental illnesses.

## INTRODUCTION

Caring for someone with a serious mental illness (SMI) is often a demanding role^1^. Caregivers need to closely monitor the patient for early signs of illness and relapse, as patients themselves are often unaware of the warning signs^2^. Caregivers also need to manage individuals’ poor functioning and erratic behavior^3^. Caring for someone with an SMI is associated with higher levels of stress than caring for someone with functional impairment due to other medical illnesses^3^. This can affect a family's overall functioning and finances and can lead to feelings of stigmatization as others judge and stereotype mental illnesses^1^. Given the negative effects of caring for a person with an SMI, there are also characteristics that make family members better equipped to cope with the challenges of caregiving. One of these characteristics is self-efficacy^2^.

Bandura defined self-efficacy as individuals’ beliefs about their ability to achieve positive results by performing an action, regardless of the difficulties and challenges related to a situation. Self-efficacy guides behavioral actions by increasing cognitive understanding, control over the situation, and self-regulatory power, and decreasing emotional reactivity to challenges. In this regard, self-efficacy determines a person's level of determination and perseverance and goal-oriented behavior in managing a particular situation^4^.

According to social cognitive theory, efficacy beliefs are multifaceted and can vary across different functional domains. Bandura stated that perceived self-efficacy scales should be tailored to the specific functioning domain of interest^5^. Accordingly, caregiver self-efficacy is defined as individuals’ belief in their own ability to perform caregiving tasks^2^. However, so far, the self-efficacy of caregivers of individuals with SMIs has been assessed only using the general self-efficacy scale^6,7^. or self-efficacy scales developed for caregivers of dementia^2,3^. Considering the difficulties of caregiving in SMIs, an appropriate tool to measure caregiver self-efficacy may be useful in this context to contribute to the care of individuals with SMIs. This study aimed to develop a scale to assess the self-efficacy of caregivers of individuals with SMIs and to determine its psychometric properties.

## METHODS

### Study procedures

#### Draft scale development

Six family members who provided care to a person with SMI of varying diagnoses were asked to recall their caregiving situations, how they overcame the difficulties they experienced while providing care, and what factors were effective in making them feel competent and successful while providing care. The recorded interview data were transcribed verbatim, and 65 statements were created regarding the self-efficacy perceptions of caregivers of individuals with SMIs in line with the interview data. Then, the literature on the self-efficacy of caregivers of individuals with SMIs was reviewed^2,3,6,8,9^, and 78 statements were created from them. A total of 143 statements were analyzed to develop the questionnaire items. As a result of discussion and evaluation by the researchers, 32 items were prepared. For these items, opinions were obtained from an expert group consisting of 14 persons who were psychiatrists and psychiatric nurses. In line with the opinions of the experts, four items were removed from the scale, two suggested items were added to the scale, and minor changes were made to eight items. The scale consists of a stem that asks a question in the "can you" format, with a 5-point response format. At the head of the items was the following instruction: "Please rate your degree of confidence in your abilities to care for a loved one with a mental illness by recording a number from 1 (cannot do at all) to 5 (highly certainly can do) using the scale provided below." The English version of this scale is a translation of the Turkish version used in this study. It was developed through discussion and back-translation by a native English translator. Therefore, it is considered semantically essentially equivalent to the Turkish version (Table 1).

**Table 1:** Items of the draft scale.

Number	Items
Item 1	Can you ensure that he/she is receiving appropriate health care?
Item 2	Can you cooperate with health-care professionals in psychiatric treatment?
Item 3	Can you maintain your daily life routine while providing care?
Item 4	Can you do anything for yourself in the time you have left after care?
Item 5	Can you think of the positive aspects of caregiving to cope with the difficulties?
Item 6	Can you try different solutions to overcome difficulties?
Item 7	Can you manage unexpected situations effectively?
Item 8	Can you cope with the negative emotions you would experience if you are stigmatized?
Item 9	Are you strong enough to be able to overcome the challenges you experience while providing care?
Item 10	Do you have enough economic potential to continue caregiving?
Item 11	Can you be motivated by the failures you experience while providing care?
Item 12	Do you think you can understand your patient?
Item 13	Can you care for him/her as long as he/she needs it?
Item 14	Do you believe you can do your best while providing care?
Item 15	Can you manage behavioral problems caused by his/her mental disorder?
Item 16	Can you manage the problems he/she experiences in his/her social relationships?
Item 17	Can you maintain a positive outlook on caregiving despite worsening symptoms?
Item 18	Do you think you can master the entire care process?
Item 19	Can you try to find a solution to his/her health problems, even if this is difficult?
Item 20	Can you make the environment you live in suitable for him/her to be comfortable?
Item 21	Are you able to handle the increased household chores due to his/her illness?
Item 22	Can your support resources enable you to maintain resilience in the face of care challenges?
Item 23	Does his/her good condition make you feel successful?
Item 24	Does it make you feel strong to receive support from your relatives in providing care?
Item 25	Can you get support for care from other family members when you need it?
Item 26	Can you follow his/her treatment plan even if it is difficult?

#### Verification of face validity

To this end, the participants were asked whether they understood the draft scale items and what the scale items emphasized. The scale was applied to 15 caregivers who were randomly selected and subsequently excluded from the sample. With the face validity verification process, the comprehensibility of the item meanings was confirmed by making minor changes in three items.

### Sample and settings

The population of the study consisted of caregivers of patients hospitalized in the psychiatry clinic of a university hospital between November 2022 and November 2023. Caregivers were included in the study who were aged 18 years and older, who were identified as primary informal caregivers by the patients hospitalized in the psychiatric clinic, who volunteered to participate, who had been providing care for the previous year, and who were caring for patients with a SMIs, such as schizophrenic disorder, schizoaffective disorder, major depression, obsessive-compulsive disorder, anorexia nervosa, and bulimia nervosa. Exploratory factor analysis (EFA) required a sample of 5–10 caregivers per item to allow for adequate inference^10^. To support a more stable analysis, 348 participants were included in the study, considering the number of items in the scale.

### Measures

Caregiver characteristics including age, gender, income level, relationship with the patient, and years of caregiving, and patient characteristics including age, gender, diagnosis, and illness duration were investigated. The 26-item draft scale was used to collect data.

### Data collection

First, potential participants were enrolled by the researchers according to the inclusion criteria. The aims of the study were then explained to the participants and their informed consent was obtained. Data were collected from caregivers accompanying patients hospitalized in the psychiatric clinic. Questionnaires with incomplete responses to any of the 26 items and those who did not meet the inclusion criteria of the study were excluded from the study.

### Data analysis

Data analysis was carried out in six stages. First, descriptive statistics were calculated for the draft scale. Accordingly, descriptive statistics including mean, standard deviation, percentage, skewness, and kurtosis were used to analyze the univariate distribution of participants’ sociodemographic characteristics and scale items.

Second, the adequacy of the sample and the suitability of the data for EFA were confirmed using Bartlett's test of sphericity and the Kaiser-Meyer-Olkin test. For an appropriate sample size, Bartlett's test of sphericity should have a significant chi-square value and Kaiser-Meyer-Olkin should have a value ≥0.70^11^.

Third, the structural validity of the scale was tested using EFA. EFA was performed using the maximum likelihood method and ProMax rotation. The number of factors was based on the Kaiser-Guttman criterion, which prescribes an eigenvalue ≥1.00^12^. The number of factors to be extracted was determined by checking the initial eigenvalue of each factor and the scree plot. Also, the items that loaded on one distinctive factor with a loading >0.45 were retained in the scale^13^. The factors generated were named.

Fourth, discriminant analysis was conducted to determine whether each item could discriminate between the highest and lowest scorers. Accordingly, the differences between the mean scores of the 27% upper and lower score groups were compared by independent sample t-test^14^.

Fifth, the reliability of the scale was tested. Cronbach's alpha was calculated for the final scale and each factor. The Cronbach's alpha value of all sub-dimensions and the total scale should be >0.70^12^. Then, corrected item–total (I–T) and item-to-item correlations were calculated. Hair et al. recommended that I–T correlations exceed 0.5 and item-to-item correlations exceed 0.3^12^.

Sixth, the test–retest method was used to evaluate the stability of the instrument across time. The scale was applied again to 32 participants 2 weeks later. The relationship between the two scores was assessed by the Spearman correlation coefficient method. The correlation coefficient value should be ≥0.70^15^.

Analyses were conducted using IBM SPSS Statistics 26 (IBM Corp, Armonk, NY, USA). In all analyses, the level of significance was set as <0.05.

### Ethical considerations

Written permission was obtained from the hospital where the research data were collected, and ethical approval was obtained from the Ethics Committee of Manisa Celal Bayar University (ID number: E-20478486-050.04.04-291391, approval date: April 20, 2022). Verbal and written informed consents were obtained from all caregivers and patients.

## RESULTS

### Characteristics of the sample

All analyses were performed on 348 caregivers. The mean age of the caregivers was 39.91±12.16 years (range: 21–66 years). Of the caregivers, 58.3% were female and 41.7% were male. In terms of income level, 23.6% had low income, 69% had income equal to expenses, and 7.5% had high income. Regarding their relationship with the patient, 11.8% of the caregivers were the patient's spouse, 25% were the patient's child, 19.3% were the patient's parent, 22.7% were the patient's sibling, and 21.3% were the patient's friend. The average caregiving duration was 6.33±6.45 years (range: 1–25 years). The mean age of the patients receiving care was 43.48±11.94 years (range: 20–67 years). Of the patients receiving care, 30.2% had schizophrenia, 5.5% had schizoaffective disorder, 38.2% had bipolar disorder, 14.4% had major depression, 6.9% had obsessive-compulsive disorder, and 4.9% had eating disorders. On average, patients had an SMI for 8.49±9.02 years (range: 1–42 years).

All items in this study were considered normally distributed except for two items. These two items were not included in further analyses. When these two items were excluded, skewness and kurtosis statistics and their associated standard errors suggested a normal distribution (skewness=-1.292 and −0.202, and kurtosis=-0.198 and 0.868, respectively) (Table 2).

**Table 2:** Analysis of draft scale items.

Items	Mean	SD	Skewness	Kurtosis
Item 1	3.48	1.07	-0.421	-0.397
Item 2	3.59	1.08	-0.602	-0.303
Item 3	3.39	1.06	-0.593	-0.245
Item 4	3.50	1.05	-0.402	-0.395
Item 5	3.35	1.07	-0.497	-0.459
Item 6	3.33	1.06	-0.525	-0.198
Item 7	3.34	1.12	-0.490	-0.465
Item 8	3.34	1.09	-0.439	-0.466
Item 9	3.31	1.09	-0.459	-0.426
Item 10	3.61	1.23	-0.618	-0.432
Item 11	3.37	1.11	-0.506	-0.534
Item 12	4.14	1.12	-1.292	0.868
Item 13	3.64	1.08	-0.512	-0.391
Item 14	3.28	1.13	-0.202	-0.765
Item 15	3.27	1.13	-0.368	-0.652
Item 16	3.35	1.20	-0.304	-0.873
Item 17	3.36	1.08	-0.567	-0.180
Item 18	3.64	1.11	-0.625	-0.254
Item 19	3.35	1.07	-0.493	-0.351
Item 20	3.59	1.15	-0.471	-0.613
Item 21	3.32	1.09	-0.466	-0.443
Item 22	3.36	1.07	-0.551	-0.289
Item 23	4.47	0.92	-2.369	5.780
Item 24	4.31	1.11	-1.857	3.741
Item 25	3.86	1.24	-1.050	0.227
Item 26	3.41	1.12	-0.131	-0.609

SD: standard deviation.

### Exploratory factor analysis and factor interpretation

Because the Bartlett test of sphericity was statistically significant (p<0.001) and the Kaiser-Meyer-Olkin index was 0.927, the data were suitable for EFA. The factor analysis was repeated by excluding items with factor loadings below 0.45. As a result, four items with a factor loading below 0.45 were removed and a three-factor scale consisting of 20 items was developed. The factors were named as the Coping Belief, the Mastery of Care, and the Persisting Despite the Difficulties of Care, respectively. The inter-factor correlation showed a significant correlation between r=0.412 and 0.450. The eigenvalue ranged from 2.258 to 7.324. The explained variance score (%) ranged from 11.289 to 36.620, explaining 59.351% of the total variance (Table 2). According to the discriminant analysis results, there was a statistically significant difference between the mean scores of each item of the upper score group (n=94) and the lower score group (n=94) (t=-39.349, p<0.001).

### Internal consistency

The I–T correlation coefficient of the scale ranged between 0.518 and 0.634 (p<0.001). Cronbach's alpha coefficients were found to be 0.917 for the overall scale, 0.914 for the Coping Belief subscale, 0.916 for the Mastery of Care subscale, and 0.883 for the Persisting Despite the Difficulties of Care subscale (Table 3).

**Table 3:** Final exploratory factor analysis of the three-factor solution for the 20-item scale with item–total correlation.

	Factor 1	Factor 2	Factor 3	EV	Explained variance (%)	I–T correlation
**Factor 1. Coping Belief** (Cronbach's alpha=0.914)				**7.324**	**36.620**	
Item 9	0.813	-0.056	-0.007			0.572
Item 3	0.805	0.087	-0.062			0.630
Item 15	0.801	-0.050	0.012			0.581
Item 21	0.778	-0.004	-0.006			0.590
Item 16	0.760	0.031	0.003			0.606
Item 7	0.739	0.075	0.003			0.622
Item 14	0.731	-0.063	0.074			0.565
**Factor 2: Mastery of Care** (Cronbach's alpha=0.916)				**2.288**	**11.441**	
Item 20	-0.069	0.897	-0.014			0.594
Item 13	0.011	0.890	-0.034			0.634
Item 18	0.001	0.847	-0.040			0.591
Item 2	-0.015	0.785	0.029			0.617
Item 1	0.013	0.767	0.070			0.610
Item 26	0.065	0.625	0.041			0.529
**Factor 3: Persisting Despite the Difficulties of Care** (Cronbach's alpha=0.883)				**2.258**	**11.289**	
Item 6	-0.013	-0.044	0.799			0.532
Item 17	-0.010	-0.037	0.792			0.533
Item 19	-0.001	-0.032	0.762			0.527
Item 11	0.006	0.028	0.698			0.518
Item 8	0.001	0.033	0.685			0.520
Item 5	0.026	0.033	0.683			0.541
Item 22	0.029	0.090	0.582			0.519
I-F correlation						
Factor 1						
Factor 2	0.411					
Factor 3	0.439	0.423				

The coefficients are statistically significant at p=0.01; EV: eigenvalue; I–T: item–total; I-F: inter-factor.

### Test–retest reliability

According to test–retest reliability, a statistically significant correlation was found for the two scores of this scale with a correlation coefficient of rho=0.856, p<0.001.

## DISCUSSION

The purpose of this study was to develop a caregiver self-efficacy scale for evaluating the self-efficacy of caregivers of individuals with SMIs and to test its psychometric properties. When the literature is examined, it is observed that this scale is the first of its kind developed to measure the self-efficacy of caregivers of individuals with SMIs^16^. EFA was conducted after confirming that the Bartlett's test of sphericity and the Kaiser-Meyer-Olkin measure met the required criteria. These results indicate that the sample size was adequate and that the data were suitable for EFA.^11^ EFA was conducted, evaluating all 24 items together. In the dimensions to which they belong, factor loads are anticipated to be high. There is a consensus that, for an item to be accepted as assessing a structure or a factor well, the minimum factor load should be 0.45^13^. It was observed that the factor loads of Item-4, Item-10, Item-12, and Item-25 were insufficient. A three-factor scale consisting of 20 items was developed. Three factors explained 59.35% of the total variance. It is acknowledged that the 40–60% variance that has been described in the literature is sufficient^17^.

Additionally, discriminant analysis demonstrated that every item could distinguish between the highest and lowest scores. As a result, all items fitted on the scale. Cronbach's alpha values were computed for the scale and all subscales for the purpose of reliability analysis. Coefficients of Cronbach's alpha greater than 0.7 are regarded as adequate^12^. In this study, the Cronbach alpha coefficients of the total scale and subscales were found to be >0.70, indicating strong internal consistency.

Another statistic that shows the reliability of a scale is the I–T correlation. In our study, the I–T correlation was positive and >0.5, supporting the internal reliability of the 20-item scale. Test–retest results for scale reliability also support consistency between repeated measurements^15^.

### Limitations

This study has several limitations that need to be considered. First, the participants were only caregivers of individuals receiving treatment at a psychiatric clinic in Turkey and only caregivers of individuals with SMIs. Considering these two limitations, it is recommended that the scale be tested in other countries and with other mental illness caregiver populations. Third, the factor structure of the scale was discovered with EFA, and hence future studies confirming the psychometric properties of this scale in different groups are needed^15^.

## CONCLUSION

According to this study, the caregiver self-efficacy scale for caregivers of individuals with serious mental illnesses is a valid and reliable instrument for evaluating the self-efficacy of caregivers of people with SMIs. The scale can evaluate the self-efficacy level of caregivers of individuals with SMIs and can be used to evaluate the results of interventions to increase self-efficacy beliefs.
